# Tackling medication errors: how a systems approach improves patient safety

**DOI:** 10.1136/ejhpharm-2025-004533

**Published:** 2025-04-25

**Authors:** Sonja Guntschnig, Renata Barbosa, Helena Jenzer, Matthew Greening, Jennifer Hayde, Helen Heery, Maria Cristina Iglesias Serrano, Kristína Lajtmanová, Elisabetta Rossin, Slagjana Tentova-Peceva, Stephanie Kohl, Alma Mulac

**Affiliations:** 1 Pharmacy, Ulster University Faculty of Life and Health Sciences, Coleraine, UK; 2 Pharmacy, Hospital da Senhora da Oliveira Guimarães, Guimaraes, Braga, Portugal; 3 Health, Bern University of Applied Sciences, Bern, BE, Switzerland; 4 Hospital Pharmacy Modernisation Team, NHS England, London, UK; 5 Pharmacy, Tallaght University Hospital, Dublin, Leinster, Ireland; 6 Portiuncula University Hospital, Ballinasloe, County Galway, Ireland; 7 NHS England, London, UK; 8 Son Espases University Hospital, Palma, Spain; 9 Hospital Pharmacy, National Institute of Cardiovascular Diseases, Bratislava, Slovakia; 10 Antiblastic Drugs Unit (UFA), ASST della Valle Olona, Nerviano, Milan, Italy; 11 Chief Hospital Pharmacist, Public Health Care Institution University Paediatric Clinic, Skopje, North Macedonia; 12 Policy & Advocacy, European Association of Hospital Pharmacists, Brussels, Belgium; 13 Oslo University Hospital, Oslo, Norway; 14 The Faculty of Mathematics and Natural Sciences, Department of Pharmacy, University of Oslo, Oslo, Norway

**Keywords:** PHARMACY SERVICE, HOSPITAL, PUBLIC HEALTH, PATIENT SAFETY, PATIENT HARM, PHARMACISTS, MEDICATION ERRORS, WORKFORCE, HOSPITALS

## Abstract

**Objectives:**

Medication errors are a leading source of preventable harm in healthcare, affecting approximately 1 in 30 patients, with a substantial proportion resulting in severe outcomes. In response, the European Association of Hospital Pharmacists convened a Special Interest Group (SIG) to propose comprehensive and sustainable strategies for reducing these errors across Europe, employing a systems approach.

**Methods:**

89 anonymised medication error reports, and empirical data from the SIG members’ daily practice, were analysed to identify root causes, classified into system-level and individual errors. Expert subgroups then linked root causes to targeted preventive measures. A literature review was conducted, searching PubMed and Embase databases, to assess existing standards and identify gaps in medication safety practices, which informed the analysis.

**Results:**

Analysis revealed that governance deficiencies and inconsistent implementation of existing legal standards contribute significantly to medication errors. System-level issues, including inadequate oversight, understaffing and insufficient technical infrastructures, along with individual errors from cognitive lapses, were prevalent. The literature review supported these findings and highlighted the variability in medication safety practices across systems, underscoring the importance of strategic improvements in healthcare policies.

**Conclusions:**

Findings highlight the critical need for robust governance, comprehensive policy frameworks and enhanced safety cultures to prevent medication errors. Automation and improved human–machine interfaces are recommended to mitigate active failures and enhance system reliability. This systems-thinking approach, supported by strengthening legislation and better resource allocation, is essential for reducing medication errors and improving patient safety.

**WHAT IS ALREADY KNOWN ON THIS TOPIC**

- Medication errors significantly impact patient safety, with existing research underscoring the need for comprehensive prevention strategies. Despite ongoing global efforts, gaps remain due to inconsistencies in safety practice implementations.

**WHAT THIS STUDY ADDS**

- This study provides a detailed systems-level analysis of medication errors, advocating for integrated safety measures, robust governance and technological enhancements like automation and human–machine interfaces to boost system reliability and prevent errors.

**HOW THIS STUDY MIGHT AFFECT RESEARCH, PRACTICE OR POLICY**

- The findings recommend a multifaceted approach to medication error prevention that could reshape research priorities, influence practice by encouraging the adoption of comprehensive safety measures and support stronger legislation and resource allocation in policy-making.

## Introduction

 Current evidence indicates that hospitalisations in low-income and middle-income countries result in approximately 134 million adverse events each year, which are associated with an estimated 2.6 million deaths annually.^1^ Globally, these adverse events pose a significant burden on healthcare systems, leading to substantial morbidity and mortality. In high-income countries, it is estimated that approximately 10% of patients experience harm during hospitalisation.^2^ The economic impact of avoidable patient harm is substantial, with the Organisation for Economic Co-operation and Development estimating that adverse events account for approximately 15% of hospital expenditures and related activities.^3^ This significant financial burden highlights the urgent need for improved patient safety measures to mitigate the occurrence of such events and optimise healthcare resource utilisation.

The WHO has initiated a series of initiatives such as the third global patient safety challenge ‘Medication without Harm’ in 2017 aimed to reduce severe avoidable medication-related harm by 50% globally by 2022,^4^ and the Global Patient Safety Action Plan 2021–2030,^5^ which addresses patient harm associated with use of medications. Several countries followed the call and began assessing the state of medication safety within their healthcare systems. The Swiss National Report on Quality and Safety in Healthcare, which included clinical reports along with contributions from various organisations found that the complexities and inconsistencies in the Swiss healthcare system require stronger governance and leadership.^6^ The conclusion that variation in pharmacy service provision needs to be reduced was a main finding of the Irish approach too.^7^ A Norwegian Critical Incident Reporting System review found that most of 3557 severe and fatal medication errors in 64 hospitals in 2016 and 2017 occurred during medication administration.^8^ A recent pan-European survey found a lack of consistency within medication error management across hospitals, with 40% reporting fewer than 100 errors annually.^9^ The WHO continued their efforts within this field recently by conducting the largest systematic review of the prevalence, severity and types of preventable medication-related harm in healthcare systems globally.^10^ The review estimated that 1 in 20 patients is affected by preventable medication-related harm and that more than one-fourth of preventable harm is severe or life-threatening.

The evidence above confirms yet again that medication errors are among the leading causes of preventable patient harm.^11^ Medication errors can occur in the entire medication management process, which starts with ordering the medication and validating orders, followed by dispensing, preparation, administration and monitoring for effects and side effects. One systematic review that focused on preventable medication harm found the highest prevalence of medication harm during prescribing and monitoring, though errors were registered across all areas.^12^ With the development of the patient safety movement, healthcare organisations have matured, and the focus from a blame approach has been shifted to promoting a just safety culture and a system-oriented approach.^13^

Despite the growing awareness of patient safety globally, only 55% of hospital pharmacies had an implemented system to ensure patient safety according to a European Association of Hospital Pharmacists (EAHP)^14^ Survey 2010 on hospital pharmacy.^14^ Although a new survey was not conducted, the EAHP continued efforts aimed at safe medication management by publishing a position paper on patient safety in 2020.^15^ The paper outlines various recommendations aimed at improving patient safety, such as closed loop medication management, medication reconciliation at transition of care and interprofessional collaboration. However, it does not specifically address the role of critical incident analysis in preventing patient harm, which is an essential aspect of understanding and mitigating medication errors.

### The accident theory and systems approach

The accident theory, particularly the Swiss Cheese Model, has significantly influenced how incident analysis is approached in healthcare. Developed by Reason, this model explains how accidents occur due to a combination of active failures (individual) and latent conditions (system-level).^16^ Healthcare systems, much like the aviation industry where the model was early adopted, are complex and comprise various layers of defence. These layers can be considered as slices of Swiss cheese, each containing holes that represent pathways for errors to occur.^17^ The approach emphasises that people are fallible, but error-prone situations can be predicted, managed and prevented. Individual behaviour is shaped by organisational culture, which is, in turn, created by leadership. Common precursors to errors include stress, time pressure, rushing, multitasking, imprecise communication, overconfidence, performing a task for the first time, distractions and interruptions. The systems approach evaluates accidents as the result of complex interactions within the system such as organisational factors, system’s design, technology, processes rather than isolated failures.^18 19^ The systems approach examines how the individual errors and system-level conditions contribute to errors.

The EAHP has established a Special Interest Group (SIG) focused on eliminating avoidable harm. The SIG was tasked to analyse current medication safety practices across Europe and develop practical strategies to reduce patient harm associated with medication errors. The work of the SIG has led to the development of recommendations designed for educational, advocacy and policy purposes, helping healthcare professionals and policymakers to implement safe medication practices and improve quality and safety in healthcare.^20^ This paper builds on the foundational work of the SIG, providing a deeper understanding of avoidable patient harm from medication errors.

The aim of this paper is to analyse, identify and describe root causes related to avoidable patient harm, particularly focusing on critical incidents and risks within the medication management process. The secondary aim was to develop measures to address the identified causes of medication errors.

## Methods

### Study design

An integrative case series design was used to answer the research question. The cases in this study consisted primarily of medication-related incident reports provided by the group members, as well as empirical data gathered from the participants’ daily work experiences. Eight SIG group members submitted 65 critical incident reports and 24 empirical data reports—all things considered, 89 incidents were discussed.

### Definition and analysis

A medication error was defined according to the National Coordinating Council for Medication Error Reporting and Prevention as ‘any preventable event that may cause or lead to inappropriate medication use or patient harm while the medication is in the control of the healthcare professional, patient or consumer’. All cases were evaluated for eligibility, and cases deemed medication errors based on the defined criteria were included in the subsequent analysis.

The analysis consisted of three stages. First, the included cases using the framework proposed by Reason were classified into:

1. System-level (latent) errors
  - Organisation and governance deficits
  - Human resources pitfalls
  - Technical resources pitfalls
2. Individual (active) errors
  - Slips and lapses
  - Mistakes

The second stage comprised identifying root causes for each case. In this stage, the SIG divided into subgroups of three to four members, where each met separately from the SIG to identify root causes. The causes were then discussed with the whole SIG. In the third stage, measures were developed to address each identified root cause using a similar approach as in stage 2. The identified root causes were linked to a concise set of actionable measures, supported by the literature review described below. These were designed to address the identified risks and prevent reported medication errors. By condensing similar recommendations into larger themes, measures to address root causes of system errors and individual errors were developed.

The summary of the results was discussed in two consensus rounds until an agreement was reached.

### Special Interest Group

13 members from 10 European countries contributed to the work of this SIG including pharmacists with expertise in medication safety and clinical pharmacy, as well as researchers experienced in analysing incident reports.

The SIG held monthly meetings for 1 year starting in March 2022. The initial phase included data collection, followed by a joint root cause analysis which was done in subgroups. The summary of the subgroup results was presented to the entire SIG, where the findings were jointly integrated.

### Literature review

A literature review of systematic reviews to synthetise the evidence relevant to the topic was conducted. The literature review was aimed to enhance the interpretation of the root cause analysis in addition to providing further evidence and supporting the development of measures to address the identified causes of medication errors. The search was performed in PubMed and Embase. The search strategy was designed to identify systematic reviews using the keywords: “medication errors”, “adverse drug events”, “patient safety” and “medication safety”. These terms were then combined with keywords: “risk factors”, “improvement”, “recommendations”, “prevent” and “measures” with AND operator. Systematic reviews and scoping reviews that focused on the impact of interventions to improve medication safety or prevent medication errors, adverse drug events (ADEs) and/or patient harm were included. Studies that described a single drug or drug class, a specific population or outpatient setting were excluded. the Covidence screening tool was used to import references, screen studies and extract data from included studies.

## Results

### Literature review

**Figure 1:**
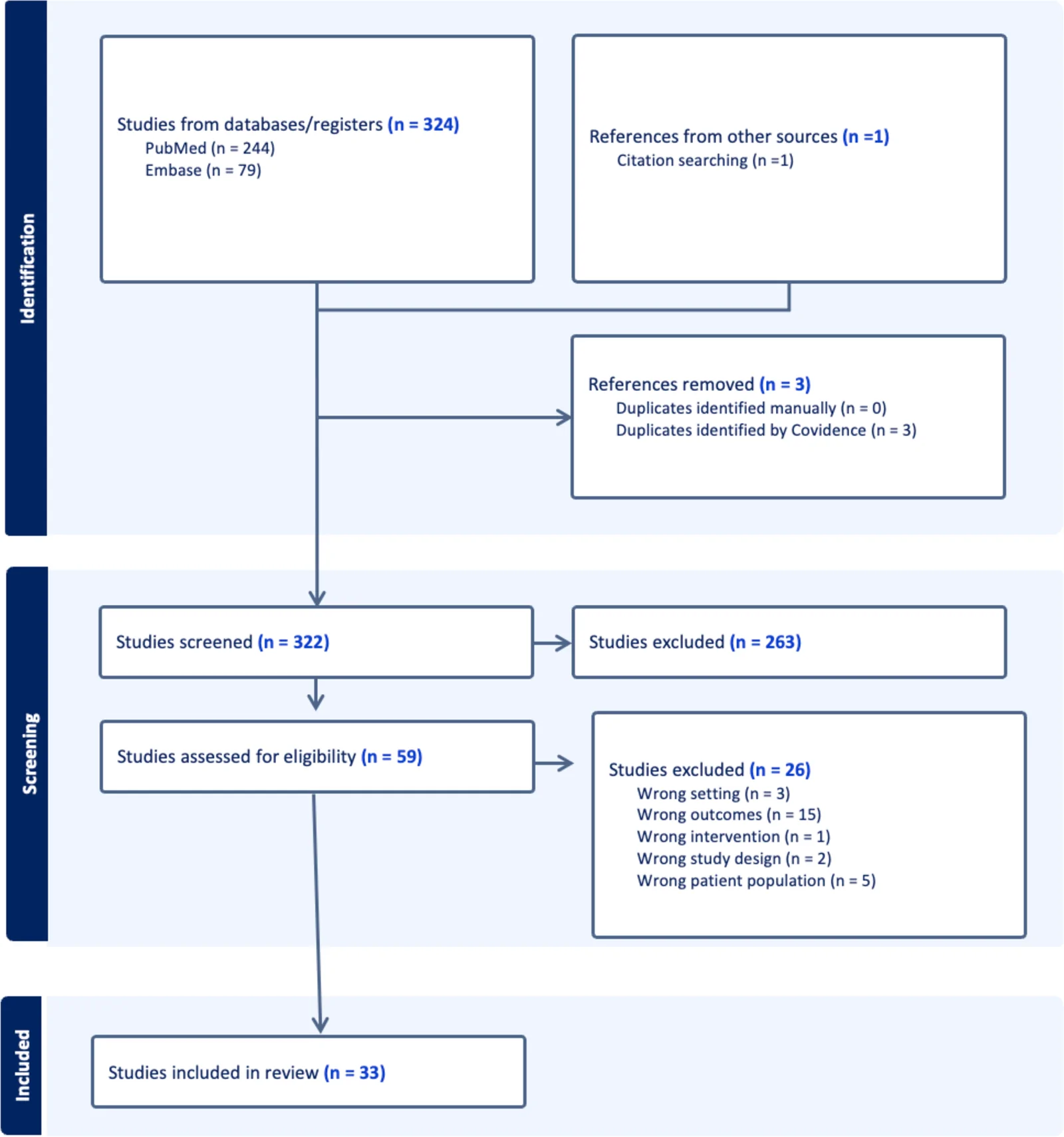
Preferred Reporting Items for Systematic Reviews and Meta-Analyses flow chart.

The literature search resulted in 324 studies of potential interest, which were screened by title and abstract. 32 studies were eligible for inclusion, and 1 study was identified from hand searching reference lists of included studies. In total, 33 reviews were found relevant to the topic. Of these, 32 studies were systematic reviews, and 1 study was a scoping review. The Preferred Reporting Items for Systematic Reviews and Meta-Analyses flow chart is presented in figure 1. The characteristics of included studies are provided in online supplemental appendix 1.

Studies were conducted across 14 countries with the majority of evidence reported from USA (n=8), UK (n=6), Canada (n=3) and France (n=3). The interventions in the included studies varied widely, with the most common being Computerised Prescriber Order Entry (CPOE) with Clinical Decision Support (CDS) (n=5)^21–25^ and Pharmacist Interventions (n=5).^26–30^ Other interventions investigated in the included studies were: CDS alone (n=3),^31–33^ double checking (n=2),^34 35^ education (n=2),^36 37^ automated dispensing cabinets (ADCs; n=2),^38 39^ patient engagement (n=3)^40–42^ and other interventions (n=9).^43–53^

### Case series

**Figure 2:**
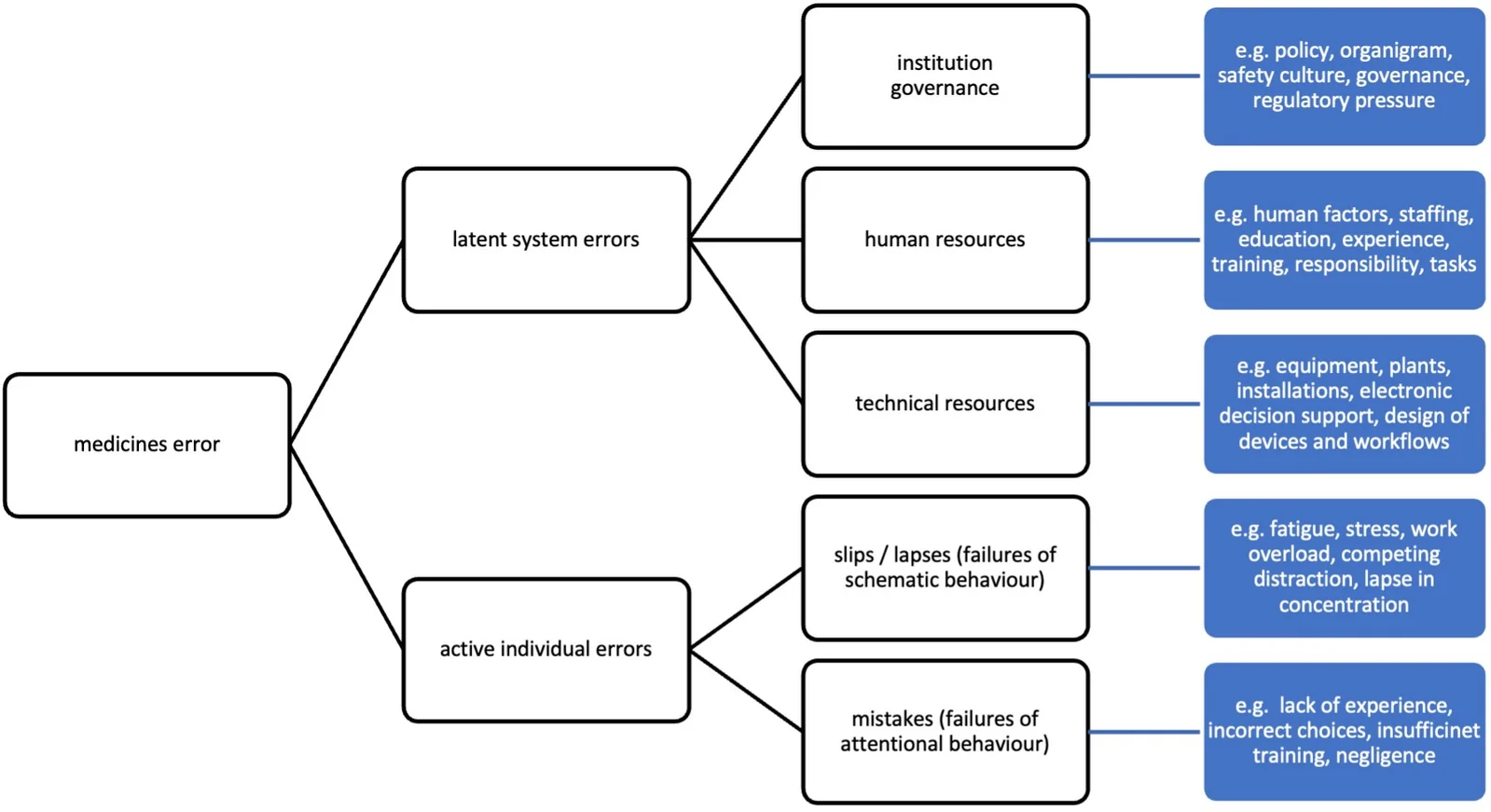
Risk identification in a multiple-step pharmaceutical supply chain.

A total of 89 cases were included in the analysis. The SIG classified the included cases using the framework proposed by Reason and listed the root causes of medication errors and harm to patients:

1. *System-level (latent) errors:* 30/89 incidents accounted for system-level errors (as classified figure 2). Their root causes were arising from organisational/governance pitfalls (7 cases), understaffed human resources (6) and underequipped technical resources (17).

- Organisation and governance deficits

Medication errors caused by organisational or governmental pitfalls include instances where responsibilities and tasks are assigned to staff members who are not qualified for those roles or cannot fully assume and share the necessary responsibilities. Error root causes are listed in online supplemental appendix 2.

- Human resources pitfalls

Medication errors caused by insufficiently qualified human resources generally lead to situations in which bad practices and deficient products or services result. Recruitment of underqualified staff and their inadequate delegation are at high risk of errors. Error root causes are listed in online supplemental appendix 3.

- Technical resources pitfalls

Medication errors caused by missing, deficient or outdated technical resources included situations in which bad practices were applied and services yielded failing performance and unsafe environment. These error root causes are listed in online supplemental appendix 4.

**Figure 3:**
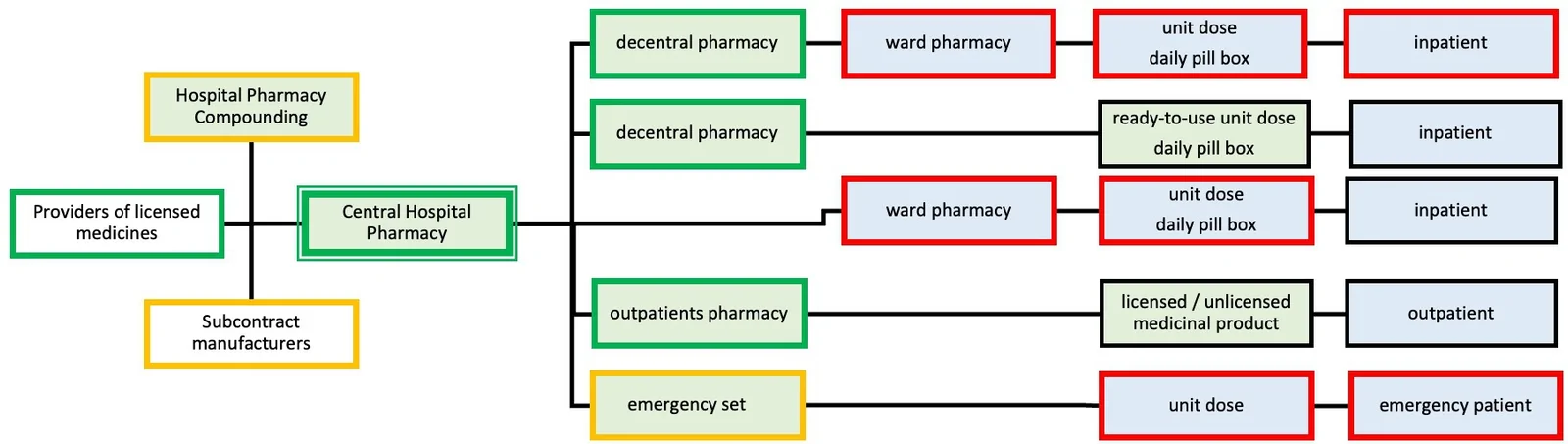
Classification of medication errors (modified from Reason 2003^17^).

1. *Individual (active) errors:* Active individual errors predominantly occurred at the points of care (see figure 3). Out of 59 identified individual errors, 8 were due to slips or lapses—errors resulting from momentary lapses in attention or memory. The remaining 51 errors were classified as mistakes, which are errors caused by incorrect decision-making or attentional lapses. Among these mistakes, 6 occurred during the supply process, 19 during order entry, prescription or dose calculation (eg, administering the wrong dose), and 26 during dose administration (eg, giving the wrong medicinal product or administering it to the wrong patient).

- Slips and lapses

Medication errors resulting from slips and lapses occurred when there were sudden deviations from routine procedures, leading to unexpected risks. These errors happened when potential failures were not anticipated and, as a result, were not addressed in a timely manner. The root causes of errors due to slips and lapses are detailed in online supplemental appendix 5.

- Mistakes

Medication errors arising from mistakes occurred in situations in which the individuals’ knowledge, expertise or skills were inadequate for coping with their tasks. These circumstances become particularly critical when high-risk parenteral medicinal products are administered. Mistakes’ error root causes are listed in online supplemental appendix 6.

## Discussion

In moving away from punitive error cultures that focus on individual blame, today’s healthcare systems emphasise anticipating and preventing errors before they occur. This shift requires addressing the root causes of systematic errors rather than solely reacting to individual incidents such as slips, lapses or mistakes^5^.

The following 11 preventive measures can be used to avoid medication-related patient harm:

### Automated drug dispensing

While a systematic review and meta-analysis^39^ found no strong evidence that automated drug dispensing systems reduce medication errors compared with manual dispensing, other research highlights potential benefits of ADCs. These include enhancing patient safety by reducing the occurrence of omitted or delayed doses, which vary in severity and could be critical. These findings underscore the importance of factors such as staffing, inventory management and system interoperability in optimising ADC performance.^38^

### Clinical Decision Support

Electronic health records (EHRs) enable the use of CDS tools to optimise medication dosing, laboratory monitoring and side effect detection. A systematic review supports the integration of test-based CDS tools in computerised physician ordering systems to manage doses, identify inappropriate medications and alert healthcare providers to crucial lab test omissions or needed treatment changes.^31^ Additionally, the variability in documenting drug allergies in EHRs poses challenges for using CDS systems effectively to screen for drug allergies, complicating the balance between reducing alert fatigue and ensuring patient safety.^32^

### CPOE combined with CDS

CPOE combined with CDS has proven effective in reducing medication error rates^22^ and preventing adverse drug events (ADEs).^25^ Systematic reviews have shown that CPOE with CDS significantly lowers medication errors in acute-care settings,^24^ although the effectiveness of specific alert types varies.^23^ Furthermore, CPOE systems improve guideline adherence and the appropriateness of alerts to some extent.^21^

### Double-checking

Double-checking procedures, especially for high-risk medications, is practiced in hospitals to reduce errors, although its overall effectiveness remains uncertain. While one study identified a significant correlation between double-checking and reduced medication errors,^35^ further research is needed to determine when and how double-checking justifies its resource requirements.

### Education and training

Education and training across all healthcare professions is key to minimising medication errors. Systematic reviews show that prescriber education effectively reduces prescription errors and associated ADEs.^37^ Similarly, nursing education on medication administration consistently yields positive, although variable, outcomes.^36^ Simulation-based learning, particularly when incorporating human factors, significantly mitigates the risks of medication errors among prescribers, nurses and pharmacy staff.^52^

### Patient engagement

Engaging patients and their families is crucial for patient safety, emphasising the need for education and medication reconciliation. Despite its importance, patient engagement frequently remains low.^40^

### Pharmacy interventions

Pharmacist-led interventions decrease the likelihood of experiencing an ADE by about a seventh compared with standard care.^28^ Including pharmacists in multidisciplinary care teams has proven effective in preventing ADEs. Multidisciplinary medication review meetings, typically involving physicians, pharmacists and nurses, are a common preventive method.^30^ Electronic medication reconciliation also significantly reduces ADE risks.^29^

### Technology

Health information technology has been critical for improving healthcare quality, particularly in enhancing adherence to guidelines, monitoring and reducing medication errors, with preventive health showing the most improvement.^47^

Various AI techniques applied across a spectrum of medications and ADEs show promise in reducing their frequency and severity through predictive analytics and early detection.^53^ Challenges with EHR interoperability are well-recognised, yet improving this can significantly enhance medication safety and reduce patient safety incidents, despite difficulties in measuring the exact impact.^50^

Data utilisation from electronic prescribing and electronic hospital pharmacy systems has generally improved medication safety, although detailed descriptions of specific interventions are scarce.^46^

### Safety of intravenous medications and smart pumps

Administering intravenous medications is inherently risky. A systematic review identified that closed-loop medication management systems, featuring smart pumps in 24% of cases, are crucial in mitigating these risks.^48^ Another review showed that while smart pumps reduce programming errors, they cannot eliminate them entirely. For maximum effectiveness, it is essential to ensure adherence to smart pump protocols, optimise drug libraries, minimise unnecessary alerts and develop strategies to reduce workarounds. Continuous quality improvement in smart pump usage is recommended to maintain their effectiveness in complex medication administration.^51^

### Overseeing risks in the medication management process

Enhancing medication error reporting is essential for data collection that supports system improvements and error prevention. Studies have shown that critical incident reporting and audit and feedback are common intervention strategies.^49^ Additionally, educational interventions and role adjustments among healthcare staff have been implemented.

In addition to the retrospective approach of analysing incident reports, risk areas can be identified through prospective risk assessments of existing and new procedures and while implementing technology. Failure mode and effects analysis is a proactive method used globally in healthcare to identify and address potential failures in the medication use process. It has proven to be an effective but time-consuming and subjective collaborative tool in enhancing medication safety.^44^

### The role of medication safety officers

Medication safety officers (MSOs), typically pharmacists by background, play a critical role in preventing medication-related patient harm by implementing proactive safety strategies within healthcare systems.^54^ These professionals identify, analyse and mitigate medication errors through system-based interventions, staff education and policy development.^55^ By integrating medication safety principles into clinical workflows, MSOs enhance adherence to best practices, reduce ADEs and foster a culture of safety.^56^ While MSOs are already well established in some European countries, their implementation remains limited or non-existent in others, leading to variability in medication safety practices.^57^ Expanding the role of MSOs across healthcare systems could standardise and improve patient safety outcomes through continuous quality improvement initiatives.

## Conclusion

Medication errors arise from both system-level and individual factors, with organisational, governance and resource-related issues playing a major role. Healthcare workers often operate under challenging conditions—limited staffing, heavy workloads and inadequate resources—which increases the risk of errors. As shown by the literature, addressing these root causes requires a comprehensive approach. Interventions such as CPOE with CDS and pharmacist-led initiatives are among the most studied. However, because intervention effectiveness varies widely, a system-wide strategy that integrates both technological and human-centred solutions is critical for reducing preventable patient harm in medication management.

In line with these findings, the SIG’s recommended measures emphasise the need to tackle organisational and governmental shortcomings (table 1). This includes reinforcing governance, nurturing a strong safety culture and creating structures that comply with existing laws and Good Manufacturing Practices. To address human resource gaps (table 2), strategies involve targeted training, workforce expansion and measures to ease workload, such as task shifting and increased automation. Updating technical resources (table 3) involves improving working conditions, decision support and supervision policies, as well as adopting health technology with user-friendly human–machine interfaces. Without these changes, active errors—whether slips, lapses or mistakes—remain likely.

**Table 1:** Measures to address root causes of system errors at the institutional level

Category	Measures to address root causes
Institutional-level measures	Assign responsibility and independent decisional power to licence-holding hospital pharmacists (see Swiss TPA, SR 812.21, art 4 and MPLO, SR 812.212.1, art 2–6) Illustrate the responsibilities of the licence holder in the organigram Define ward pharmacies as part of the hospital pharmacy Implement a blaming- and punishment-free safety culture Standardise PICS PE 010-4 guidance (‘GMP guide for healthcare establishments’) Abolish all quality stipulations not released by the license holder Standardise CAPA (corrective and preventive actions) to correct quality deviations Implement medicines reconciliation and medication review

GMP, Good Manufacturing Practices.

**Table 2:** Measures to address root causes of system errors at the human resource (HR) level

Category	Measure to address root causes
HR level measures	Allocate sufficient qualified HR (for staffing disparities, see HIQA: https://www.hiqa.ie/reports-and-publications/key-reports-and-investigations/medication-safety-monitoring-programme) Limit work overload, time pressure and staff fatigue Exclude unqualified staff from preparing and administering medicines Shift tasks from overburdened nursing staff to pharmacy technicians and clinical pharmacists

**Table 3:** Measures to address root causes of system errors at a technical resources level

Category	Measures to address root causes
Revising working conditions	Subordinate all preparation activities to the production-license holder ((CM/Res[2016)1)and(CM/Res[2016)2)) Stop critical processes if 4-eye principles cannot be applied Design distraction- and interruption-free workplaces for critical handling and high-risk medicines
Automating processes	Optimise communication and information flow Synchronise pharmacy and clinical information systems Predefine electronic workflows Standardise man–machine interfaces Eliminate manual transcribing (see *CycloGESTCytoTECA2019.pdf* (imsn.ie) for electronic prescribing and order-entry with plausibility testing) Implement clinical decision support systems Invest in robotic systems and electronic dispensing cabinets Introduce barcode labelling and bedside scanning

Ultimately, governance bodies, authorities, regulators and hospital leaders must resolve these systemic flaws through effective oversight and equitable resource distribution. Establishing a just culture that prioritises proactive error prevention over blame is vital. Moreover, actively involving patients in safety efforts helps safeguard them from harm and builds more resilient healthcare systems.

## Supplementary material

10.1136/ejhpharm-2025-004533 online supplemental file 1

## Data Availability

No data are available.
