# Supplementary material for: Tackling medication errors: how a systems approach improves patient safety
Source: Eur J Hosp Pharm. 2025 Apr 25;33(5):e004533. doi: 10.1136/ejhpharm-2025-004533 (PMC13539831; doi:10.1136/ejhpharm-2025-004533)
Supplement: Supplementary file 1 [file ejhpharm-33-5-s001.docx]

| **Author, year** | **Country** | **Title** | **Measure theme** |
| --- | --- | --- | --- |
| Jeffrey, 2024 | UK | Automated dispensing cabinets and their impact on the rate of omitted and delayed doses: A systematic review | Automated drug dispensing |
| Carvalho, 2020 | Brazil | Effectiveness of the automated drug dispensing system: systematic review and meta-analysis |  |
| Whitehead, 2019 | USA | The Effect of Laboratory Test-Based Clinical Decision Support Tools on Medication Errors and Adverse Drug Events: A Laboratory Medicine Best Practices Systematic Review | Clinical Decision Support (CDS) |
| Légat, 2018 | Belgium | Clinical Decision Support Systems for Drug Allergy Checking: Systematic Review |  |
| Bayoumi, 2014 | Canada | The effectiveness of computerized drug-lab alerts: a systematic review and meta-analysis |  |
| Wolfstadt, 2008 | Canada | The effect of computerized physician order entry with clinical decision support on the rates of adverse drug events: a systematic review | Computerized Prescriber Order Entry with CDS |
| Radley, 2013 | USA | Reduction in medication errors in hospitals due to adoption of computerized provider order entry systems |  |
| Page, 2017 | Australia | A systematic review of the effectiveness of interruptive medication prescribing alerts in hospital CPOE systems to change prescriber behaviour and improve patient safety |  |
| Kaushal, 2003 | USA | Effects of computerized physician order entry and clinical decision support systems on medication safety: a systematic review |  |
| Eslami, 2008 |  | The impact of computerized physician medication order entry in hospitalized patients--a systematic review |  |
| Alsulami, 2012 | UK | A systematic review of the effectiveness of double checking in preventing medication errors | Double checking |
| Koyama, 2020 | Australia | Effectiveness of double checking to reduce medication administration errors: a systematic review |  |
| Härkänen, 2016 | Finland | Systematic review and meta-analysis of educational interventions designed to improve medication administration skills and safety of registered nurses | Education |
| Bos, 2017 | The Netherlands | The effect of prescriber education on medication-related patient harm in the hospital: a systematic review |  |
| Kim, 2018 | USA | Evaluation of Patient and Family Engagement Strategies to Improve Medication Safety | Patient engagement |
| Richardson, 2014 | UK | Evaluating the effectiveness of self-administration of medication (SAM) schemes in the hospital setting: a systematic review of the literature |  |
| de Lusignan, 2014 | UK | Patients' online access to their electronic health records and linked online services: a systematic interpretative review |  |
| Zaij, 2023 | France | Intervention of pharmacist included in multidisciplinary team to reduce adverse drug event: a qualitative systematic review | Pharmacist Interventions |
| Killin, 2021 | Canada | Advanced Medication Reconciliation: A Systematic Review of the Impact on Medication Errors and Adverse Drug Events Associated with Transitions of Care |  |
| Kelly, 2024 | USA | Association of Pharmacist Interventions With Adverse Drug Events and Potential Adverse Drug Events |  |
| Chiewchantanakit, 2020 | Thailand | The effectiveness of medication reconciliation to prevent medication error: A systematic review and meta-analysis |  |
| Alhmoud, 2024 | Qatar | Impact of pharmacist-supported transition of care services in the Middle East and North Africa: a systematic review and meta-analysis |  |
| Syrowatka, 2022 | USA | Key use cases for artificial intelligence to reduce the frequency of adverse drug events: a scoping review | Other interventions |
| Sarfati, 2019 | France | Human-simulation-based learning to prevent medication error: A systematic review |  |
| Ohashi, 2014 | USA | Benefits and risks of using smart pumps to reduce medication error rates: a systematic review |  |
| Li, 2022 | UK | The Impact of Electronic Health Record Interoperability on Safety and Quality of Care in High-Income Countries: Systematic Review |  |
| Kuitunen, 2021 | Finland | Systemic Defences to Prevent Intravenous Medication Errors in Hospitals: A Systematic Review |  |
| Gleeson, 2020 | Ireland | Interventions to improve reporting of medication errors in hospitals: A systematic review and narrative synthesis |  |
| Chaudhry N. T., 2021 | UK | The Secondary Use of Data to Support Medication Safety in the Hospital Setting: A Systematic Review and Narrative Synthesis |  |
| ChaudhryB., 2006 | USA | Systematic review: impact of health information technology on quality, efficiency, and costs of medical care |  |
| Berdot, 2016 | France | Interventions to reduce nurses' medication administration errors in inpatient settings: A systematic review and meta-analysis |  |
| Anjalee, 2021 | Sri Lanka | Application of Failure Mode and Effect Analysis (FMEA) to improve medication safety: a systematic review |  |
| Acheampong, 2014 | Ghana | Medication safety strategies in hospitals--a systematic review |  |

Appendix 1 Characteristics of studies included in the literature review

| **Examples of institution’s and governance’s latent system’s error root causes** | |
| --- | --- |
| Category | **Error Root Causes** |
| **Governance Deficits** | - Lack of legally binding standards (explicitly GMP guidance) outside hospital pharmacy - Unstandardized or lacking quality assurance systems on wards - Lack of functioning Drug & Therapeutics Committee - Lack of hospital formulary including medicines for special patient groups - Lack of safety culture and medication safety policy - Lack of adequate corrective and preventive action as follow-up of errors - Lack of adequate risk management for high-risk injectables |
| **Incomplete Organisation Chart** | - The responsibility for the entire pharmaceutical supply chain is not assigned. - The licence holder’s responsibility and authority to issue directives is not illustrated in the enterprise organigramme. - Ward pharmacies are not subordinated to the license-holding hospital pharmacy. - Manufacturing or dispensation activities of medicinal products are not GMP compatible and are performed outside of the supervision of the licence holder |
| **Medicines Reconciliation and Medication Review** | - Not implemented or performed by unqualified persons |

Appendix 2 Examples of institution’s and governance’s latent system’s error root causes

| **Examples of human resources latent system’s error root causes** | |
| --- | --- |
| Category | **Error Root Causes** |
| **Inadequate Human Resources Allocation** | - Excessive workload - High error risk due to wrong delegation of tasks to personnel not apt to respond to requirements and not trained. - Misuse of the qualified person’s compounding and manufacturing license without supervision |
| **Staffing Disparities** | - No national or international standard to ensure adequate quality of various services. - Lack of knowledge and risk management skills (both enablers of high quality) - Gaps in curriculum leading to high-risk situations. - Dry recruitment market: Employment of unqualified personnel as substitutes for qualified professionals during staffing shortages |
| **Communication Errors** | - Personnel unable to understand professional jargon |
| **Lack of Training Time** | - Insufficient time for training and learning on the job |

Appendix 3 Examples of human resources latent system’s error root causes

| **Examples of technical resources’ latent system’s error root causes** | |
| --- | --- |
| Category | **Error Root Causes** |
| **Communication & System Disruptions** | - No state-of-the-art communication - Frequent system disruptions inducing extra costs - Poor qualification standards: Not fully documented functionality of infrastructure, fixed/mobile installations, and equipment - High risks due to bad design of working environments - Contamination of working environments and products during construction or renovation over working hours |
| **Ill-Conceived Men-Machine Interface** | - Misuse of non-personal IT logins and/or passwords - Poor menu captions leading to dosing errors:    - Omitted or double dispensing    - Misleading units (drops, mL, mg, mcg, µg)    - Inadequate medicine substitution - Manual data entry affecting compliance with the 5R rules (right patient, right medicine, right dose, right route, right time)    - Lack of barcode support    - Missing status updates on patient doses (prepared, controlled, distributed, dispensed) - Missing or poorly designed interfaces between main and supporting information systems (patient administration, medication stock/logistics, medical records, context-driven pop-ups) - Over-alerting in clinical decision support systems - Poor parameterization (e.g., physical unit errors) combined with slow or inadequate IT support, troubleshooting, and upgrades - Excessive time loss for change management |
| **Lack of Automation** | - Lack of box-picking machines, medication dispensing systems, and blistering machines, leading to supply chain and manual dose preparation errors    - Pill organizers too small for prescribed medicines    - Re-used pill organizers potentially cross-contaminated    - Unblistered medicines preventing patient identification of own medication    - Lack of ready-to-use liquid unit doses - Lack of networked electronic prescribing systems and barcode technology    - Confusion due to look-alike/sound-alike medications    - Failures in patient and product identification    - Undetected decimal floating-point errors (10.0 vs. 1.00)    - Persistence of:     - Illegible handwriting     - Fallible double-check/4-eye principle/5R rules     - Hard-to-trace paper-based systems (e.g., manual control of controlled substances) - Lack of therapeutic drug monitoring leading to missed detection of toxic plasma levels due to insufficient equipment |

Appendix 4 Examples of technical resources’ latent system’s error root causes

| **Root causes of individual (active) Errors – examples of slips and lapses** | |
| --- | --- |
| Category | Error root causes |
| **Slips & Lapses** | - Patient-owned medicinal products left in the bedside drawer - Undetected development of an addiction - Undetected hypernatraemia - Reordered doses adapted only for one day instead of continuously - Modified prescription after the discharge letter had already been edited - Failure to open clamp on an elastomeric pump |

Appendix 5 Root causes of individual (active) Errors – examples of slips and lapses

| **Root causes of individual (active) errors - examples of mistakes' root causes** | |
| --- | --- |
| Category | Error Root Causes |
| **Storage-Level Mistakes** | - Unqualified/inexperienced workers in a wholesaler’s warehouse unable to recognize cracks in dropped infusion bags - Multiple failures of the 4-eye principle:    - Confounding of sound-alikes and look-alikes    - Information gap related to imported medicinal products |
| **Prescription / Order Entry / Dose Calculation Errors** | - No interaction check performed - Undetected allergy history against a medicinal product - Omitted double-check leading to:    - Undetected underdosing    - Omitted loading dose    - Erroneous insulin unit administration    - Undetected overdosing    - Inversion of weight and height    - Bolus injection instead of diluted solution injection    - Confounding of physical units (time vs. mg)    - Prescription of wrong infusion speed    - Inappropriate fluid volume and/or infusion speed    - Harmful fluid retention due to sodium overload |
| **Dose Administration / Dispensing / 5R Rule Errors** | - Wrong medicine (e.g., ropivacaine infusion instead of acetaminophen infusion) - Omitted or extra doses administered - Medicine given to the wrong patient - Treatment of non-responders - Wrong patient/wrong doses administered (often by a factor of 10 or 100) - Administration of unlabelled syringes to wrong patients - Crushing of slow-release galenic forms - Wrong amounts of liquid medicines (drops, mL, mg) - Wrong route of administration    - Omeprazole for nasogastric line connected to the central intravenous line    - Concentrated infusion of 40 mmol potassium chloride given through a peripheral line instead of a central line - Wrong time (e.g., TPN bag running too long) - Erroneous picking of look-alikes (similar presentations of different dosages) - TPN compounded with an electrolyte-containing component instead of electrolyte-free - Confounding of anti-D immunoglobulin and varicella immunoglobulin - Confounding of insufficiently labelled p.o. and i.v. syringes - Infusion pump running with swapped lipid and aqueous syringes due to near-identical protective covers, leading to incorrect infusion rates |
| **Lack of Medicines Reconciliation, Medication Review, & Seamless Care** | - Undetected double dosing of opiates on patient admittance - Multiple dosing errors following internal patient transfer - Undetected adverse reaction of ciprofloxacin in a patient with epilepsy - Several preadmission medications omitted - Neglected pharmacokinetics and pharmacodynamics in a cardiology patient (leading to heart failure) |
| **Revising Working Conditions** | - Subordinating all preparation activities to the production-license holder ([CM/Res[2016)1] and [CM/Res[2016)2]) - Stopping critical processes if 4-eye principles cannot be applied - Designing distraction- and interruption-free workplaces for handling high-risk medicines |
| **Automating Processes** | - Optimizing communication and information flow - Synchronizing pharmacy and clinical information systems - Predefining electronic workflows - Standardizing men-machine interfaces - Eliminating manual transcribing (e.g., using electronic prescribing and plausibility testing) - Implementing clinical decision support systems (CDSS) - Investing in robotic systems and electronic dispensing cabinets - Introducing barcode labelling and bedside scanning - Addressing missing, unqualified, or untraceable medicines reconciliation |

Appendix 6 Root causes of individual (active) errors - examples of mistakes' root causes

Appendix 1 Characteristics of studies included in the literature review

Appendix 2 Examples of institution’s and governance’s latent system’s error root causes

Appendix 3 Examples of human resources latent system’s error root causes

Appendix 4 Examples of technical resources’ latent system’s error root causes

Appendix 5 Root causes of individual (active) Errors – examples of slips and lapses

Appendix 6 Root causes of individual (active) errors - examples of mistakes' root causes
